# A qualitative systematic review on AI empowered self-regulated learning in higher education

**DOI:** 10.1038/s41539-025-00319-0

**Published:** 2025-05-03

**Authors:** Min Lan, Xiaofeng Zhou

**Affiliations:** 1 Zhejiang Key Laboratory of Intelligent Education Technology and Application, Zhejiang Normal University, Jinhua, 321004 Zhejiang China; 2 College of Education, Zhejiang Normal University, Jinhua, Zhejiang Province China

**Keywords:** Education, Human behaviour

## Abstract

This systematic review explores the burgeoning intersection of Artificial Intelligence (AI) applications and self-regulated learning (SRL) in higher education. Aiming to synthesize empirical studies, we employed a qualitative approach to scrutinize AI’s role in supporting SRL processes. Through a meticulous selection process adhering to PRISMA guidelines, we identified 14 distinct studies that leveraged AI applications, including chatbots, adaptive feedback systems, serious games, and e-textbooks, to support student autonomy. Our findings reveal a nuanced landscape where AI demonstrates potential in facilitating SRL’s forethought, performance, and reflection phases, yet also highlights whether the agency is human-centered or AI-centered leading to variations in the SRL model. This review underscores the imperative for balanced AI integration, ensuring technological advantages are harnessed without undermining student self-efficacy. The implications suggest a future where AI is a thoughtfully woven thread in the SRL fabric of higher education, calling for further research to optimize this synergy.

## Introduction

The recent advancements in Artificial Intelligence (AI) applications, such as large language models like ChatGPT, have opened new avenues for enhancing teaching and learning processes in higher education^1^. According to Salas-Pilco et al.^2^, AI can significantly enhance student learning performance, engagement, and interest, particularly in STEAM (Science, Technology, Engineering, Arts, and Mathematics) disciplines. For instance, advanced learning analytics and intelligent assistants, which support students by offering real-time feedback and personalized learning pathways, can promote deeper understanding and retention of course material^3^.

Despite the promising opportunities, several challenges hinder the widespread and effective adoption of AI in higher education. From a technological aspect, insufficient digital literacy or AI literacy among students and educators poses significant obstacles^4^. From a pedagogical aspect, integrating AI into complex educational systems requires careful consideration to avoid undermining academic integrity and ensuring high-quality outputs^5^. Zawacki-Richter et al.^6^ emphasized the weak connection between AI applications and theoretical or pedagogical frameworks in higher education. However, many studies fail to incorporate underlying educational theories into their AI-enabled processes^7^.

Therefore, there is a pressing need for integrating educational and learning theories into AI-driven teaching and learning^1^. As suggested by Chu et al.^8^, higher education practitioners must stay abreast of the latest AI developments to effectively leverage these tools for enhancing instructional practices. We should focus on empirical investigations that examine the practical effects of AI applications in real-world educational scenarios, moving beyond technological aspects to explore their impact on teaching and learning processes^1,9^. Therefore, AI applications require learners’ (both teachers and students) self-regulation for the utilization in their learning and instruction, especially for teachers, as their agency for technology use in their teaching may significantly impact students’ learning effectiveness and performance^10^.

By examining the theoretical considerations of self-regulated learning (SRL), this study seeks to provide a comprehensive understanding of how AI technologies can support and enhance educational practices in higher education, through a qualitative systematic review. While the SRL frameworks rooted in traditional educational contexts have established substantial theoretical and practical implications, the role and functions of AI within SRL processes remain underexplored. Therefore, guided by established SRL frameworks, this study aims to examine how AI-enabled tools influence and potentially reshape these frameworks. The findings from this review will offer valuable insights for educators, researchers, and policymakers, guiding future implementations and research in the rapidly evolving field of AI in education. Four research questions (RQs) are outlined below to guide this study:

**RQ1**: What are the characteristics and contexts of studies that explore the use of AI tools in supporting SRL?

**RQ2**: What theories underpin the studies on AI-assisted SRL, and how do these theories explain the connection between AI tools and the SRL process?

**RQ3**: What specific AI applications are being utilized to enhance SRL, and in what ways do these applications support students in managing their learning processes?

**RQ4**: Based on empirical evidence, what are the observed benefits and potential drawbacks of using AI applications in supporting SRL?

## Results

**Fig. 1:**
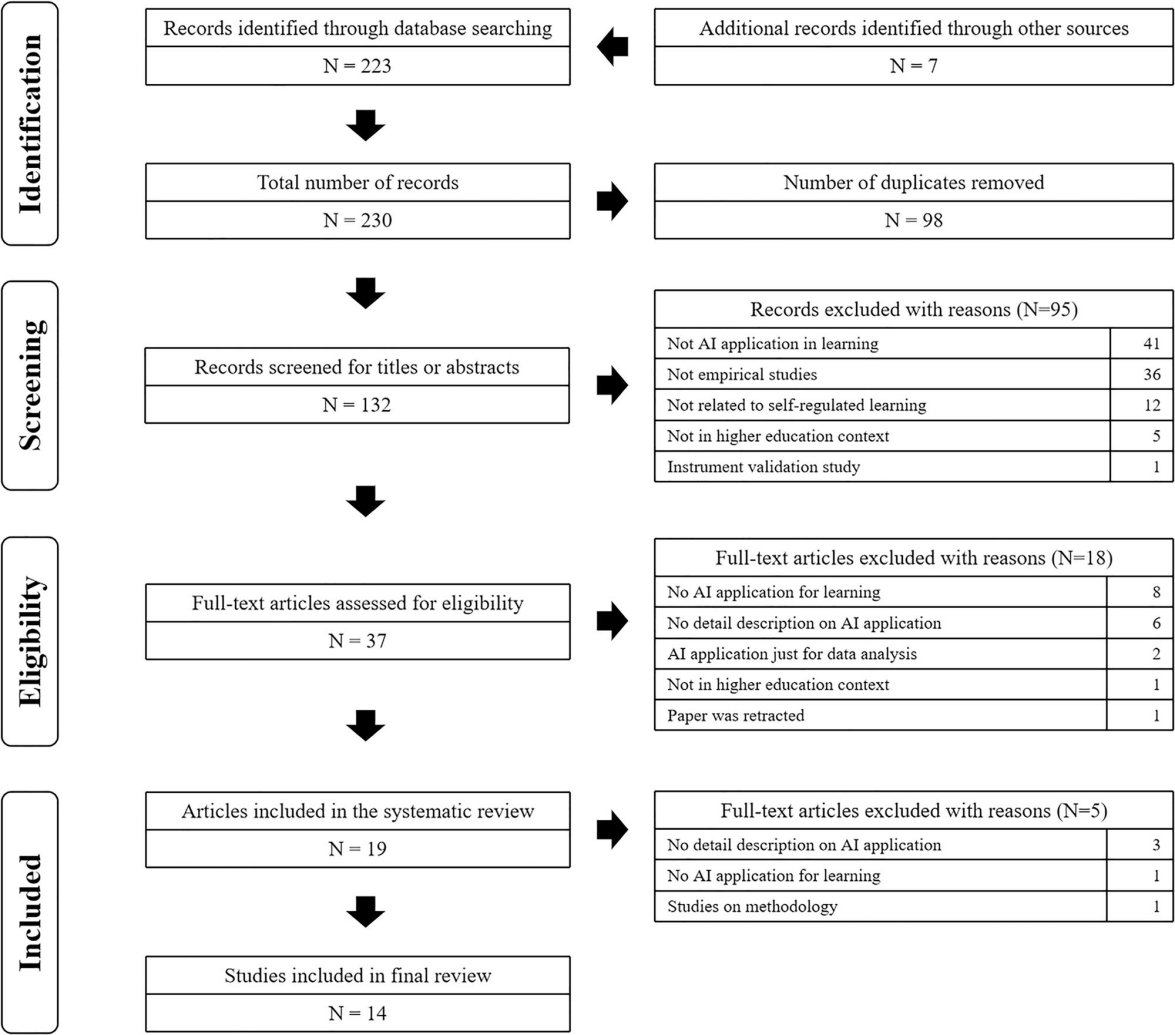
Preferred Reporting Items for Systematic Reviews and Meta-analyses (PRISMA) flow chart. The process of article screening encompasses the following stages: identification, screening, eligibility, and inclusion. The identification stage involves the results of searching databases based on keywords and the screening of articles from article references and referenced articles. Following the removal of duplicates, the screening process was initiated to identify articles that did not meet the eligibility criteria. This involved a two-step approach: first, the titles and abstracts of the articles were browsed, and then the full texts were read. Articles that were deemed to potentially meet the eligibility criteria were selected, and the full texts were again browsed to identify any articles that did not meet the eligibility criteria. The selected articles were then sorted and analyzed.

A total of 230 articles were found from the database services and relevant reference lists in the first round of identification. After removing 98 duplicates, 132 articles were identified. After reviewing the titles and abstracts, 37 full-text articles were found to be eligible for further review. Of these, 18 articles did not meet the inclusion criteria and were excluded. Of the remaining 19 articles, five did not meet the inclusion criteria and had to be excluded. Hence, a total of 14 distinct studies were identified for qualitative. Figure 1 presents the PRISMA flow chart summarizing the results of our processes on identification, screening, and article selection.

### Basic information about these studies

14 studies investigated the application of AI to support students’ SRL (see Table 1 for details). Most examined the effectiveness of AI tools in enhancing learning outcomes, with some also exploring the mechanisms that contribute to their success and areas for improvement. Research methodologies varied: 6 (42.86%) studies used quantitative experimental designs to test the impact of AI on learning, 7 (50%) employed mixed-method approaches to both evaluate outcomes and understand underlying perceptions and only one (7.14%) utilized process mining. The studies spanned a range of contexts: 7 (50%) focused on STEM subjects (e.g., biology, distributed systems, digital systems, and computer science), 5 (35.71%) on language learning (reading and writing), and two (14.29%) on non-academic areas (communication skills and exam anxiety). Geographically, the research was diverse, covering different regions globally.

**Table 1:** Basic information on the studies reviewed

Study	Research aim	Research design	Results summary
Yin et al.^28^	Investigating the impact of a chatbot-based micro-learning system on students’ learning motivation and performance	The research method used in this study is a quasi-experimental design. **Sample and sample size**: first-year university students (*N* = 99) **Learning context**: Conversion of Numerical Systems **Data collection**: Test and motivation instrument **Data analysis**: Descriptive analysis and ANCOVA test **Location**: China	The study showed that chatbot-based micro-learning matched traditional teaching in terms of student performance but significantly increased intrinsic motivation due to perceived choice and value. This approach was especially effective for students who preferred having autonomy in their learning process.
Afzaal et al.^29^	Developing a data-driven feedback and intelligent action recommendation (DFIAR) dashboard based on the proposed explainable ML-based approach. This study was to evaluate the utility and limitations of the developed dashboard.	The research method used in this study is mixed-method design. **Sample and sample size**: university students (*N* = 314) **Learning context**: Programming **Data collection**: exam scores, motivation questionnaire, perceived choice questionnaire, perceived value questionnaire. **Data analysis**: descriptive statistics, paired t-tests, ANOVA, correlation analysis, regression **Location**: Sweden	The study found that chatbot-based micro-learning matched traditional methods in student performance but significantly boosted intrinsic motivation by offering more choice and value. This approach was particularly effective for students who prefer autonomous and flexible learning environments.
Koc-Januchta et al.^15^	Exploring the impact of using an AI-enriched digital biology textbook on university students’ learning experience, focusing specifically on the differential effects of three sub-types of cognitive load: intrinsic, germane, and extraneous.	The study utilized a mixed-methods research approach. **Sample and sample size**: university students (*N* = 42) **Learning context**: introductory biology course **Data collection**: surveys at various points, measuring cognitive load, learning strategies, self-regulation, and usability, along with pre-and post-course knowledge tests **Data analysis**: Quantitative analysis included descriptive statistics, t-tests, and a GLM Repeated Measures procedure, with Pillai’s trace to ensure robust ANOVA results. Qualitative analysis was conducted on students’ open-ended responses on usability. **Location**: United States	Findings suggested a positive learning pattern where students experienced significantly higher levels of germane cognitive load compared to intrinsic and extraneous loads, indicating they were consistently engaged in meaningful learning activities. A significant correlation was observed between the use of the AI book’s feature that offered linked suggested questions and a higher germane load, implying the tool promoted deep learning. However, some students reported usability issues that negatively impacted learning gains, likely due to design aspects that diverted cognitive resources away from productive learning processes. Despite this, the overall sentiment towards the AI-book was positive.
Mai et al.^19^	Examining the impact of different interaction methods employed by a coaching chatbot—specifically, click-based versus writing-based interactions—on the working alliance formed between the chatbot and human coaches. This is particularly focused on the context of student coaching addressing exam anxiety.	This is an exploratory quantitative study. Two distinct chatbot systems were developed: one utilizing a click-based interface where users select from predefined responses (implemented on a rule-based system), and another featuring a writing-based interface enabling free-text input (powered by conversational AI). **Sample and sample size**: engineering students (*N* = 21) **Learning context**: exam anxiety **Data collection**: a survey to assess the working alliance and chat histories **Data analysis**: Statistical analysis **Location**: Germany	The results indicate that the working alliance in both study conditions can be classified as medium to high overall. The results further show higher values for bonding on a writing-based platform than when using a click-based system. However, click-based systems seem to be more helpful as a low-threshold entry point to coaching, as they guide coaches better through the process by providing predefined answers. An evaluation of the technical realization shows that self-reflection processes through digital self-coaching via chatbot are generally well accepted by students.
Fidan & Gencel^30^	Investigating the effects of artificial intelligence (AI)-based chatbot and peer feedback mechanisms integrated into the instructional videos as a feedback tool on learning performance and intrinsic motivation of pre-service teachers in online learning	The research method used in this study is Mixed method experimental design. **Sample and sample size**: pre-service teachers (*N* = 144) **Learning context**: science and social science **Data collection**: exam scores, motivation questionnaire, perceived choice questionnaire, perceived value questionnaire **Data analysis**: descriptive statistics, paired t-tests, ANOVA, correlation analysis, regression analysis **Location**: Turkey	Fidan’s study found that supporting instructional videos with chatbot interactions significantly enhanced students’ learning outcomes and engagement. Students who used the chatbot showed higher quiz scores and reported greater satisfaction and motivation compared to those who only watched the videos. The chatbot’s ability to provide immediate feedback, answer questions, and guide learners through the content contributed to these improved educational experiences.
Zhang & Xu^17^	Investigate how ethnic minority students engage with automated writing evaluation (AWE) systems in their academic writing process.	The research method used in this study is a qualitative approach. **Sample and sample size**: ethnic minority students (*N* = 12) **Learning context**: academic writing **Data collection**: multiple sources including student multiple-draft assignments, AWE feedback, and retrospective interviews **Data analysis**: Textual analysis **Location**: China	The participants were found to engage with the AWE system behaviorally, cognitively, and affectively in their writing and revision, but the extent to which they engaged in the process is influenced by their multilingual proficiency, familiarity with digital technologies, learning beliefs and sociocultural identities. Those who were more actively engaged and obtained more learning gains tended to capitalize on their linguistic and cultural capital in their engagement with AWE feedback in their writing.
Zhang & Hyland^31^	Examining the effectiveness of an integrated feedback approach in promoting students’ behavioral, affective, and cognitive engagement with feedback on their writing and encouraging thoughtful revisions.	The research method used in this study is a case study. **Sample and sample size**: third-year Chinese university student (*N* = 33) **Learning context**: language learning **Data collection**: demographic information, pre-test scores, post-test scores, engagement and interaction metrics, survey responses on chatbot experience, qualitative feedback from interviews **Data analysis**: descriptive statistics, paired t-tests, ANOVA, thematic analysis of qualitative data **Location**: China	Zhang’s study revealed that integrating comprehensive feedback mechanisms significantly boosted student engagement and performance. By combining automated feedback, peer reviews, and teacher assessments, students experienced higher motivation and deeper involvement in their learning activities. The diverse feedback methods helped clarify learning objectives, provided immediate insights into performance, and encouraged reflective learning practices. This integrated approach led to improved academic outcomes and a more engaging and responsive learning environment.
Al-Abdullatif et al.^18^	Evaluating the impact of implementing the Bashayer chatbot, an AI-based system, on the motivation and learning strategies of postgraduate students in Saudi higher education.	A quasi-experimental design was employed. The experimental group then interacted with the Bashayer chatbot through WhatsApp for 13 weeks, while the control group continued with conventional learning methods. **Sample and sample size**: postgraduate students (*N* = 60) **Learning context**: Reading in Educational Technologies in English Language and Dissertation **Data collection**: Motivated Strategies for Learning Questionnaire (MSLQ). **Data analysis**: comparing the pre-and post-test scores to measure changes **Location**: Saudi	The findings indicated that students in the experimental group, who used the Bashayer chatbot, exhibited higher levels of both cognitive and meta-cognitive learning strategies compared to the control group.
Afzaal et al.^32^	Investigating how informative feedback and explainable AI-based recommendations can facilitate students’ SRL, aiming to improve their academic performance in a data-driven manner.	This is a mixed-method study. **Sample and sample size**: undergraduate (356) **Learning context**: programming course **Data collection**: academic achievement and self-regulation skills **Data analysis**: t-tests, ANOVA, and correlation analysis, as well as thematic analysis of survey responses **Location**: Sweden	The results revealed that the dashboard significantly enhanced students’ learning achievements and improved their SRL skills. Furthermore, it was found that the recommendations generated by the proposed approach positively affected students’ performance and assisted them in self-regulation.
Hare et al.^16^	Assessing the impact of the PING system, an integrated intelligent tutoring system (ITS) within a serious game (SG), on student engagement and learning outcomes in digital logic education.	A quantitative pre/post-experimental research methodology was employed. Participants were randomly assigned to either a standard lab without a game, an older version of the game Gridlock, or the new version of Gridlock featuring full personalized support from the PING system. **Sample and sample size**: public university students (*N* = 109) **Learning context**: (1) Introduction to Digital Systems and (2) Computer Architecture **Data collection**: pre/post-tests on vending machine design and surveys measuring students’ attitudes towards the game and its learning environment. **Data analysis**: Game log analysis, pre-/post-test analysis **Location**: United States	The results showed that students who interacted with the PING-enhanced game reported higher content knowledge, quicker completion times, fewer errors, and increased efficiency in problem-solving. They also expressed positive attitudes towards the game, finding its tools useful and easy to use, particularly the error diagnosis feature. Student surveys and game log data supported these findings, indicating a balanced blend of learning and enjoyment fostered by the game’s design.
Kuzminska et al.^33^	Exploring the potential of artificial intelligence (AI) tools in enhancing the group dynamics of self-organized teams working on educational projects, focusing on their impact on self-efficacy and student engagement.	This is a mixed-method study. Based on the results of the free choice of project and team management tools, we categorized three groups among the 56 participants of the experiment according to the use or non-use of AI tools to enhance group dynamics, followed by self-assessment of the effectiveness of the selected tools in increasing their levels of self-efficacy and social engagement. **Sample and sample size**: 4th year students of the National University of Life and Environmental Sciences of Ukraine (Software Engineering educational program) (*N* = 56) **Learning context**: Group Dynamics and Communications **Data collection**: survey **Data analysis**: non-parametric methods of analysis and statistical hypothesis testing **Location**: Ukraine	The self-assessment confirmed that the incorporation of AI tools in the implementation of a team-based learning project did not influence the improvement of students’ social engagement and the dynamics of self-efficacy for both effective and ineffective students. The authors identified the positive impact of using AI tools on its development for students with the average level of self-efficacy.
Li & Kim^34^	Alleviating the challenge faced by self-regulating English learners in higher education, particularly international students, who struggle with identifying areas for improvement in their language skills autonomously.	The study is a mixed method study, which primarily employed a qualitative approach to understand the students’ preferred AFS tools and critical engagement throughout their personalized learning journeys but it also included a small-scale quantitative component. **Sample and sample size**: international students from diverse backgrounds (*N* = 32) **Learning context**: language learning **Data collection**: students’ e-portfolios, focus group interviews as well as a survey **Data analysis**: thematic analysis, comparative statistical analysis **Location**: Australia	Results indicate positive perceptions and successful utilization of AFSs by students, who leverage these tools to pinpoint improvement areas, monitor progress, and boost confidence. The study emphasises the importance of course structure, teacher guidance and a combination of human and automated feedback, in fostering learner autonomy and emotional self-regulation.
Nguyen et al.^20^	Examining the nature of human-AI interactions in academic writing, specifically investigating the strategies doctoral students employ when collaborating with a GAI-powered assisting tool.	This study applied process mining techniques to analyze human-AI collaboration in an academic writing task. **Sample and sample size**: doctoral students (*N* = 10) **Learning context**: academic writing **Data collection**: writing log-file records and questionnaire **Data analysis**: data pre-processing and analysis with quantitative content analysis, sequence analysis with Hidden Markov Model (HMM) and hierarchical sequence clustering, and pattern analysis with process mining **Location**: Finland and New Zealand	Findings indicate that doctoral students engaging in iterative, highly interactive processes with the GAI-powered assisting tool generally achieve better performance in the writing task. In contrast, those who use GAI merely as a supplementary information source, maintaining a linear writing approach, tend to get lower writing performance.
Ortega-Ochoa et al.^35^	Analyzing the effectiveness of empathic chatbot feedback for developing computer competencies, motivation, self-regulation, and metacognitive reasoning in online higher education.	A quasi-experimental design was used. in which a conversational agent, DSLab-Bot, was integrated into the syllabus and Information Technology infrastructure. **Sample and sample size**: diverse background in an online higher education institution (*N* = 196) **Learning context**: Distributed Systems course **Data collection**: the practice assignments test and questionnaire (processing of feedback, cognitive feedback, and affective feedback, motivation, self-regulation, metacognitive reasoning **Data analysis**: descriptive and inferential statistics, non-parametric Mann–Whitney U test **Location**: Spain	Results showed no significant differences between the groups in learning performance, motivation, or self-regulation, except in one item of motivation (self-efficacy) and self-regulation. There were strong correlations between thirteen cognitive and seven affective chatbot feedback types with conceptual change (MRCC) and personal growth and understanding (MRPGU). There were high weights of similar chatbot feedback types indicating a pronounced influence of these on meta-cognitive reasoning components, even self-reflection (MRSR).

The following table provides a concise overview of the research aim, research design, and result for each article that has been reviewed.

### Theoretical foundation

Among the learning explanation theories (see Table 2 for details), SRL theory emerges as the most prominent, featuring in 4 studies (28.57%). Other significant theories include self-determination theory (2 studies, 14.29%), cognitive theory (2 studies, 14.29%), and engagement theory (2 studies, 14.29%). Specifically, the four studies that utilized SRL theory predominantly relied on the models proposed by Zimmerman, Pintrich, and Winne. These models emphasize the processes of learning and the critical influence of feedback from behavioral, cognitive, and affective perspectives. Even when SRL models were not directly applied, several studies aligned their conceptual frameworks with SRL processes. For instance, engagement theory, which similarly addresses the behavioral, cognitive, and affective dimensions of learning, demonstrates a significant theoretical alignment with SRL. Cognitive theory also emphasizes the strategic selection and management of cognitive processes by learners, aligning closely with the tenets of SRL. Furthermore, self-determination theory highlights intrinsic motivation, closely linked to the self-motivational beliefs in the forethought phase of SRL. This connection extends to the monitoring and control behaviors during learning, as well as the learner’s capacity for reflection.

**Table 2:** The theoretical foundation on the studies reviewed

Types of foundation		Theory	How to relate SRL
Theory for learning explanation	Cognitive Load Theory	Cognitive Load Theory^36^ focuses on the mental effort, or cognitive load, imposed on working memory during task execution^37^. It identifies three types of cognitive load: intrinsic (ICL), extraneous (ECL), and germane (GCL). Intrinsic cognitive load (ICL) arises from the complexity of the task itself and the cognitive activities required to understand the inherent information. Extraneous cognitive load (ECL) occurs when learners expend mental resources on non-essential aspects of the task. Germane cognitive load (GCL) is associated with the construction of schemas or mental models during meaningful learning processes.	**[Performance phase]:** Cognitive load is strongly connected with self-regulated learning. For example, high cognitive load might originate from students’ insufficient self-control skills and low willingness to learn^38^. Self-regulated learners are more likely to improve their academic achievements by selecting and controlling cognitive processes involved in learning^39^.
	Cognitive process of writing	The cognitive process of writing, as outlined by Flower & Hayes^40^, involves intensive mental effort^41^ required for tasks such as generating ideas, maintaining coherence, organizing content, and adapting it to fit the intended audience or context. Writing tasks utilize the limited capacity of working memory^42,43^, and if cognitive demands exceed this capacity, writing performance can suffer^44^. Flower & Hayes categorize the writing process into planning, translating, and reviewing stages, all of which are influenced by the writer’s working memory capabilities and the specific demands of the task environment^40^.	**[Performance phase]:** Becoming a proficient writer involves improving performance through deliberate effort and skillful control over the writing process^45^. Effective management of writing activities is crucial for producing high-quality texts^46^. Skilled writers demonstrate strong regulation abilities, shifting from sequential to concurrent strategies to avoid overwhelming their working memory capacity^47^. This adaptive approach helps maintain coherence and efficiency in their writing.
	Engagement theory	Fredricks, Blumenfeld, and Paris^48^ define student engagement as a meta-construct comprising three dimensions: behavioral, emotional, and cognitive. Behavioral engagement involves active participation in school activities, task involvement, and positive conduct. Emotional engagement relates to students’ feelings of belonging, interest in learning, and affective responses to academic content, peers, and teachers. Cognitive engagement encompasses students’ psychological commitment to learning, their use of learning strategies, and their ability to self-regulate during academic tasks.	**[Forethought phase]:** Affective engagement focuses on students’ emotional and attitudinal reactions to Automated Writing Evaluation (AWE) feedback. **[Performance phase]:** Cognitive engagement involves the use of cognitive strategies and revision techniques in response to this feedback. **[Self-reflection phase]:** These findings indicate that the effectiveness of AWE feedback depends on how students engage with it during writing and revision processes.
	Self-determination theory	The self-determination theory^49^ posits that intrinsic motivation—driven by inherent desires such as enjoyment, interest, curiosity, and satisfaction—is a critical factor influencing an individual’s cognitive, social, and physical development within learning processes. This natural motivational tendency is considered more significant than extrinsic motivation, which is influenced by external factors like rewards and coercion. The theory emphasizes that activities performed due to inherent interest are crucial for the advancement of knowledge and skills.	**[Forethought phase]:** SDT emphasizes the importance of intrinsic motivation in the initial phase of SRL, where learners set goals and plan their learning activities. Intrinsic motivation, as described by SDT, can lead to more effective goal setting and planning because learners are engaged due to personal interest and satisfaction in the learning process. **[Performance phase]:** During the execution of learning activities, SDT’s concept of intrinsic motivation aligns with SRL by encouraging learners to persist and engage deeply with the material. **[Self-reflection phase]:** SDT suggests that intrinsic motivation can lead to more positive self-reflection, as learners are more likely to attribute their successes to their efforts and abilities, rather than external rewards. This reflection can further enhance intrinsic motivation and self-regulation in subsequent learning activities.
	Self-regulated learning theory	According to Zimmerman’s self-regulated learning model^50,51^, SRL comprises three primary phases: (i) the forethought phase, where students engage in pre-action thinking including goal setting, strategic planning, motivation, and building self-efficacy; (ii) the performance phase, focused on implementing plans, monitoring study progress, and managing time effectively; and (iii) the reflection phase, which involves evaluating learning actions and engaging in self-reflection on the learning process.	**[Forethought phase]:** In the forethought phase, it assists students in setting course goals and planning their learning activities accordingly using Algorithm 1. Recommendations generated align tasks with the set goals, prioritizing them and assigning required scores to enhance motivation. **[Performance phase]:** During the performance phase, the dashboard categorizes recommendations into assignments, lecture tasks, videos, and exercises, allowing students to mark tasks completed and receive new ones as they progress. It also provides progress monitoring, highlighting areas where students are falling behind and indicating the percentage completed. **[Self-reflection phase]:** In the reflection phase, the dashboard predicts students’ final exam performance based on completed actions, encouraging self-reflection and adjustment of learning strategies. It also evaluates completed recommendations, identifying those not meeting requirements to prompt further action and completion.
		This study utilizes Pintrich’s framework^52^ to assess the impact of the Bashayer chatbot system on motivational orientation, specifically focusing on task value, self-efficacy, and cognitive and metacognitive learning strategies.	**[Performance phase]:** After integrating Bashayer with the teacher’s WhatsApp number, it was managed through the Freshdesk platform. This setup allowed the teacher to communicate with students directly using their own Freshdesk dashboard. When students interacted with Bashayer via WhatsApp, their queries were routed to Freshdesk, where the system’s database was accessed to find relevant answers. **[Self-reflection phase]:** The responses were then sent back to students through Bashayer via WhatsApp, completing the communication loop facilitated by the Freshdesk-hosted platform.
		According to Butler and Winne^53^, the recursive nature of Self-Regulated Learning (SRL) highlights that feedback plays a crucial role in stimulating learners’ regulation of subsequent cognitive engagement.	**[Forethought phase]:** Offering feedback on the learners’ language goal. **[Performance phase]:** Offering feedback on precise activities aimed at attaining their goal. **[Self-reflection phase]:** Offer feedback on the disparity between the learner’s goal and their current level.
Theory for learning design	Instructional model	During the stages of group development proposed by Tuckman and Jensen^54^ — forming, storming, norming, performing, and adjourning — students maintained observation journals and generated self-reporting reports.	**[Forethought phase]:** Forming, where members acquaint themselves and may struggle with task understanding. Storming is characterized by pushing beyond initial boundaries; Norming, where differences are resolved and strengths recognized. **[Performance phase]:** Performing is when the group works smoothly towards goals without conflict. Adjourning is marked by disengagement, anxiety over separation, and closure.
		The coaching process in this study, based on Berninger-Schäfer’s framework^55^, consists of three phases: onboarding, situation description and clarification, and closure.	**[Forethought phase]:** The chatbot’s onboarding process is crucial and involves welcoming the user, introducing itself, and explaining its tasks to establish expectations and boundaries^56,57^. It also requests the user’s name for more personalized interaction. **[Performance phase]:** Following onboarding is the situation description and clarification phase, beginning with a scale question to gauge the user’s exam anxiety level from 1 to 10. This question helps to identify if the user fits the coaching target group. Specific systemic coaching questions are then used to explore and clarify the situation, including problem-oriented questions that illustrate how problems are created and perpetuated, and solution-oriented questions like the miracle question (“How would you know your exam anxiety is gone?”). **[Self-reflection phase]:** The chatbot provides mini tips on managing exam anxiety, such as self-organization techniques or breathing exercises. The interaction concludes with a goodbye phase.
	Teaching strategies	1. Divide-and-conquer^58^: This method involves breaking down complex tasks into smaller, more manageable parts to facilitate easier resolution or completion. Repetition^59^: It emphasizes the practice of repeating tasks or information multiple times to enhance learning and retention. Problem-based learning^60^: This approach fosters learning through the exploration and resolution of real-world problems, promoting critical thinking and application of knowledge. Gamification^61^: Incorporating game design elements into non-game contexts like education to enhance engagement, motivation, and learning outcomes.	**[Performance phase]:** Serious games (SGs) and Intelligent Tutoring Systems (ITS) assess and support student performance. Repetition in tasks reinforces learning, with varied quizzes and backgrounds enhancing engagement. SGs also immerse students in practical problem-solving^60^ while gamified elements^61^ maintain engagement and focus on educational content.
		Chatbot-Mediated Learning by doing^62^ integrates interactive chatbot technology into the Distributed Systems syllabus to enhance educational interventions. This methodological strategy combines hands-on learning with Chatbot-Mediated Learning (CML), aiming to enrich the overall educational experience through practical application.	**[Forethought phase]:** This integration was designed to enhance practical learning outcomes that align with the course schedule. **[Performance phase]:** The DSLab-Bot, available on Mattermost within DSLab, served as a learning tool for this methodological strategy, actively utilized during assignments. **[Self-reflection phase]:** DSLab-Bot was integrated into the Practice Assignments Phases, emphasizing its role in providing feedback during interactions.

The following table summarizes the theoretical foundation of the reviewed papers. It enumerated the nomenclature of the theories, their respective explanations, and the manner in which these theories pertain to the concept of self-regulated learning (SRL).

In terms of design frameworks, the studies highlight the application of instructional models and teaching strategies, providing practical insights for the design of AI applications in education. Instructional models offer sequential guidance for both designers and educators, delineating the steps to be implemented at each stage of the instructional process. Teaching strategies, conversely, provide recommendations at the activity level, enabling AI tools to effectively target specific learner behaviors, emotions, and cognitive actions. These frameworks not only facilitate the integration of AI in educational settings but also enhance the precision and efficacy of AI tools in addressing the nuanced needs of learners.

### What and how AI application facilitate learning

The reviewed studies utilized four types of AI applications: chatbots (7 studies, 50%), evaluation systems (5 studies, 35.71%), serious digital games (1 study, 7.14%), e-textbooks (1 study, 7.14%, see Table 3 for details). Chatbots were the most frequently used. These studies leveraged chatbots to facilitate writing-based natural human communication for help-seeking, feedback, and learning monitoring and management. 5 out of them integrated AI tools into existing platforms, such as WhatsApp, a learning management system, or a dashboard platform. The remaining two studies did not design or develop new tools but explored the use of existing AI applications based on students’ preferences. AI-empowered evaluation systems were also frequently used. Four of these systems targeted language learning, providing language assessment and feedback on programming language. Only one study developed a system to evaluate students’ SRL processes and offer recommendations for learning adjustments. To support domain-specific learning, two studies designed a serious digital game and an e-textbook, respectively, for subjects like math and biology. These tools provided clear content and scenarios, enabling students to seek help throughout their learning journey.

**Table 3:** AI-empowered learning application from the perspective of self-regulated learning

Types of application	Study	What AI application	How does this AI application support self-regulated learning
Serious Game	Hare et al.^16^	A domain-specific serious game called Gridlock integrates a problem-focused approach to educating students in digital logic design. **[Backend Technology]:** Gridlock integrates with a reinforcement learning (RL) engine known as the personalized instruction and need-aware gaming (PING) system. RL is an AI technique that uses trial and error to explore different approaches to a problem, learning to determine the optimal behavior through this process.	**[Performance phase]:** (1) The system employs repetition and quizzes to reinforce concepts **(Self-control)**; (2) The system provides automated assistance during activities and assessments **(Help-seeking)**.
Evaluation system	Afzaal et al.^29^	A learning analytics technique combined with explainable machine learning to provide automatic and intelligent feedback and action recommendations that support students’ self-regulation. **[Backend Technology]:** The study presents a machine learning (ML) process for predicting student performance and generating feedback. It involves data preprocessing from an LMS, feature extraction, and addressing class imbalances. Predictive models are built and refined using cross-validation. The best model uses explainable ML to identify key performance factors and provide feedback.	**[Self-reflection phase]:** (1) This system offers data-driven feedback and tailored action recommendations through a dashboard, empowering students to self-regulated learning **(Self-evaluation)**.
	Afzaal et al.^32^	An explainable AI-based approach to provide automatic and intelligent feedback and recommendations that can support the self-regulation of students’ learning in a data-driven manner. **[Backend Technology]:** The study involved three key steps: (1) Feature generation grouped into six categories: quiz, assignment, exercise, activity completion, lecture activity, and video lecture attributes. (2) Predictive model building and evaluation using six machine learning algorithms (logistic regression, k-nearest neighbors, support vector machine, random forest, artificial neural network, and BayesNet) to predict students’ academic performance in their final exams. (3) Generation of recommendations with explainable AI, where a counterfactual explanation algorithm (Algorithm 1) used the best-performing model to provide personalized, actionable recommendations for each student.	**[Forethought phase]:** (1) The dashboard helps students set course goals and plan their activities, providing prioritized recommendations and required scores to achieve these goals **(Goal setting and strategic planning)**; (2) Throughout the course, it evaluates completed tasks and updates recommendations to guide students effectively **(Goal setting)**. **[Performance phase]:** It categorizes tasks such as assignments and lecture videos, allowing students to take action and track their progress, highlighting areas needing improvement **(Self-monitoring and self-control)**. **[Self-reflection phase]:** The dashboard predicts final exam performance, motivating students to reflect on their learning behaviors and adjust accordingly **(Self-judgment)**.
	Li & Mira^34^	Different mobile- or computer-assisted automated feedback systems (AFSs) for language learning assessment. **[Backend Technology]:** Not clarify	**[Forethought phase]**: (1) In the PELE context, diagnostic tools like the PELE questionnaire, ELSA, Grammarly, and PaperRater assist learners in selecting and refining their goals. The PELE questionnaire suggests broad objectives such as pronunciation or academic writing **(Goal setting)**; (2) Learners are guided to use a combination of these tools to gain comprehensive diagnostic insights across goal setting, gap analysis, and targeted practice **(Goal setting and task analysis)**. **[Performance phase]:** Tools like ELSA and MaiMemo track progress to boost motivation **(Self-monitoring)**. **[Self-reflection phase]:** (1) ELSA, Grammarly, and PaperRater offer detailed feedback to focus on specific areas within these goals **(Self-evaluation)**; (2) To measure the gap between current abilities and goals, tools like vocabulary tests, Speech Rate Meter, and VoiceAnalyst are used **(Self-judgment)**.
	Zhang & Hyland^31^	The AWE system, Pigai, generates feedback on texts comprising real-time holistic scoring, diagnostic feedback, and overall end comments through language processing technologies and statistical algorithms. **[Backend Technology]:** Not clarify	**[Self-reflection phase]:** Students submitted their first drafts to the Pigai AWE system for immediate feedback and subsequently revised their work based on the received suggestions, resulting in their second drafts **(Self-evaluation)**.
	Zhang & Xu^17^	Automated writing evaluation (AWE) systems, Pigai, a web-based English writing evaluation program, offers corrective feedback, holistic scoring, highest and lowest scores, and overall comments. **[Backend Technology]:** Not clarify	**[Self-reflection phase]:** Pigai offers corrective feedback, holistic scoring, highest and lowest scores, and overall comments. It generates an overall score for an essay by calculating its quantitative differences from the texts in its corpora in four dimensions: vocabulary, sentence, organization, and relevance **(Self-evaluation)**.
E-textbook	Koc-Januchta et al.^15^	AI-enriched digital biology textbook that integrates a 5000-concept knowledge base and algorithms offering the possibility to ask questions and receive answers. **[Backend Technology]:** Specific AI elements applied in the AI-enriched book comprise a formal knowledge representation of book content, natural language processing to interpret a student’s inputted or selected suggested questions, and natural language mechanisms for generating answers.	**[Performance phase]:** (1) The AI-enriched book also offers a 5000-concept knowledge base and algorithms that provide the possibility to ask questions and receive answers **(Self-monitoring and help-seeking)**; (2) Natural language processing to interpret a student’s inputted or selected suggested questions **(Help seeking)**.
Chatbot	Mai et al.^19^	Rasa is a conversational AI, which is used to recognize the users’ intentions in order to enable the writing-based interaction method. **[Backend Technology]:** In Rasa, conversational AI enables dialogue through Natural Language Understanding (NLU), which converts user speech into structured files by interpreting messages as intents (like greetings or agreements). This process uses machine learning libraries such as Tensorflow and spaCy. High accuracy in predicting conversation flow requires numerous possible user responses within these intents. Keywords in intents can also be defined as entities, like recognizing a user’s name. The core of Rasa’s chatbot functionality is Natural Language Processing (NLP), a machine learning category that converts and interprets free text, making sense of user input by considering chat histories and identifying key information.	**[Forethought phase]:** During onboarding, the chatbot introduces itself, explains its capabilities, and asks for the user’s name to personalize interactions **(Goal setting and task analysis)**. **[Performance phase]:** Users can receive tips on managing exam anxiety, such as self-organization or breathing exercises **(Help-seeking and self-control)**. **[Self-reflection phase]:** The chatbot then uses solution-oriented questions to explore how users might recognize the reduction of their anxiety **(Self-evaluation)**.
	Al-Abdullatif & Al-Dokhny^18^	A task-oriented chatbot, that is embedded into the social learning platform via WhatsApp application, called Bashayer. **[Backend Technology]:** The Freshdesk platform hosts and manages the Bashayer chatbot system, integrating it into social media apps like WhatsApp. It facilitates text-based interactions between students and the chatbot, allowing teachers to use Freshdesk’s dashboard to respond to inquiries, access database information, and manage chatbot functions.	**[Performance phase]:** (1) Students’ inquiries sent through WhatsApp were processed by Freshdesk, which searched its database for answers and replied to students through Bashayer **(Help-seeking)**; (2) Students can access an education consultant **(Help-seeking)**. (3) The tool provides a course progress summary **(Self-monitoring)**.
	Ortega-Ochoa et al.^35^	A conversational agent, DSLab-Bot, was integrated into the syllabus and Information Technology infrastructure. **[Backend Technology]:** The AIoT network’s main components include software solutions, with DSLab-Bot at its core, and physical hardware like laptops and PCs used by students. DSLab-Bot integrates into the DSLab IT infrastructure via the open-source tool Mattermost and the university’s Single Sign-On Authentication system, enabling private message notifications and automated user registration through Mattermost’s REST API. The Java-based BotEngine intercepts messages and responds to student queries using the RASA tool, which employs a TensorFlow-based core to classify and generate responses. This setup is supported by a dedicated server and a MySQL database that stores question-answer pairs and emotional evaluations. The DSLab-Bot’s emotional capabilities are powered by a fuzzy logic classifier that interprets the emotional weights of words to enhance interactions.	**[Performance phase]:** (1) To respond to questions **(Help-seeking)**; (2) To detect emotions **(Self-monitoring)**. **[Self-reflection phase]:** (1) To give feedback on the outcome of project execution in the distributed environment **(Self-evaluation)**; (2) To receive positive or negative student feedback on its response **(Self-judgment)**.
	Nguyen et al.^20^	Any generative AI-powered assisting tool during academic writing. **[Backend Technology]:** The initial integration of AI-driven applications, such as grammar and style checkers, to enhance written work quality is well-documented. With advancements in natural language processing (NLP), AI tools have evolved to offer extensive support in writing. Generative AI (GAI) technologies, like GPT-3 and GPT-4, now enable content generation, summarization, feedback, and improvement suggestions. These advanced tools assist writers throughout the writing process, from idea generation to drafting and revising.	**[Performance phase]:** Participants were given 30 minutes to complete the writing task, during which they were allowed to use any tools deemed necessary, including ChatGPT and Google Scholar **(Self-control)**.
	Kuzminska et al.^33^	not designed, decided by the students themselves, mainly focused on ChatGPT and Notion AI. **[Backend Technology]:** Not clarify	**[Performance phase]:** Students decided what AI tools they could use, mainly focused on ChatGPT and Notion AI **(Self-control)**.
	Yin et al.^28^	Students interacted with the chatbot-based micro-learning system autonomously to acquire knowledge. **[Backend Technology]:** The chatbot-based micro-learning system was developed using Google Dialogflow for managing intents (learning topics) and entities (contextual terms). Machine learning interprets user queries, matches them to intents, and retrieves relevant content from a knowledge base. The chatbot was integrated with Microsoft Bot Framework to create a streamlined web chat interface, chosen for its simplicity. Students accessed the chatbot via a provided link for easy use on their devices.	**[Performance phase]:** As students engage, they can ask questions and receive immediate, contextually relevant responses that guide them through specific learning checkpoints. This interactive flow allows the chatbot to provide instant feedback, helping students to reinforce their knowledge and monitor their progress efficiently (**Help-seeking and self-monitoring**).
	Fidan & Gencel^30^	The chatbot “Bilge” was designed theoretically to respond to pre-service teachers in the way of elaborate and personalized feedback. **[Backend Technology]:** PVA integrates multiple AI models centered around a transformer-based Natural Language Understanding (NLU) core. This deep neural network supports functions like machine translation, chat transcript mining, interpretable ML, context handling, dialogue management, automatic suggestions, and reinforcement learning.	**[Self-reflection phase]:** Elaborated and provide immediate computer-generated feedback **(Self-evaluation)**.

The following table summarizes the types of applications in the reviewed papers, including the specific AI application in each type, the backend technology description, and the manner in which the AI application supports self-regulated learning (SRL) based on Zimmerman’s three-phase SRL model.

**Fig. 2:**
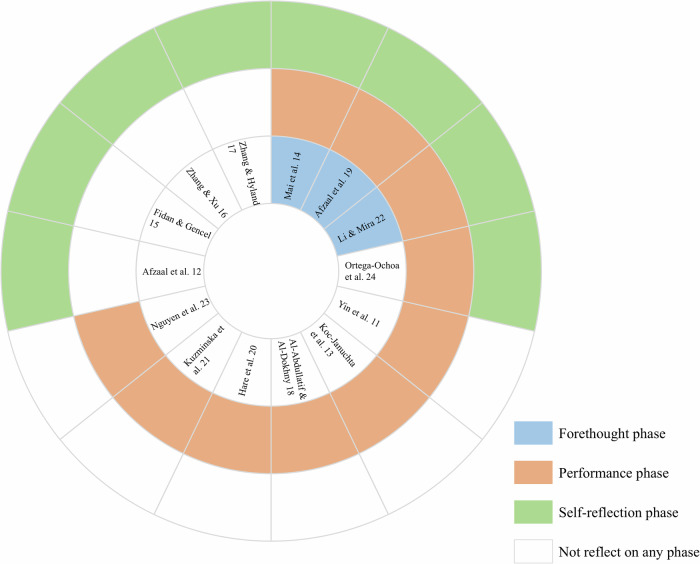
How the AI applications support self-regulated learning processes based on Zimmerman’s three phases SRL model. Based on the three-phase SRL model, each AI application or tool will be investigated which phase have they facilitated. In this figure, the blue color, orange color, and green color mean the AI application or tool in that study has facilitated the forethought phase, performance phase, and self-reflection phase in SRL correspondingly. The white color means there is no support for the AI-enabled SRL process.

**Fig. 3:**
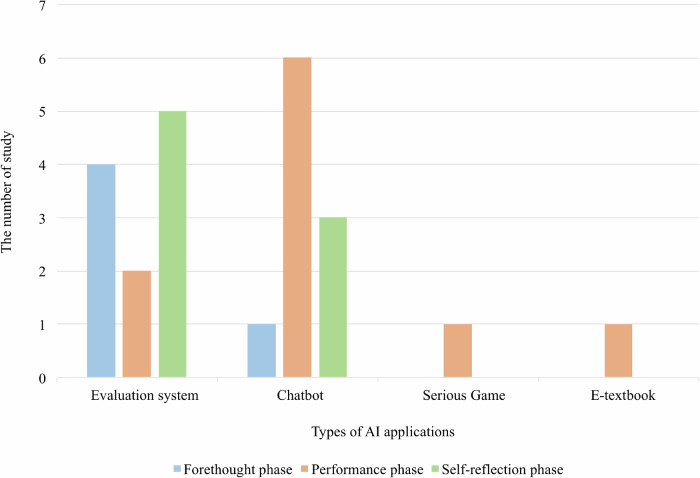
How different types of AI applications support self-regulated learning processes based on Zimmerman’s three phases SRL model. Based on the three-phase SRL model, each AI application or tool will be investigated which phase have they facilitated. These applications or tools have been categorized into four groups. In this figure, the blue color, orange color, and green color mean the AI application or tool in that study has facilitated the forethought phase, performance phase, and self-reflection phase in SRL correspondingly.

To varying extents, all the AI applications in the study facilitated students’ SRL (See Fig. 2). However, only three (3 of 14) applications provided comprehensive support across all phases of SRL as outlined in Zimmerman’s model, in which three phases are aligned. Half of these studies (7 of 14) concentrated their support on the performance phase (focusing on help-seeking, learning monitoring and instructional strategies). Four studies (4 of 14) merely focused on the support for the self-evaluation phase (emphasizing providing feedback for self-reflection). Notably, only one application was dedicated exclusively to supporting SRL processes. In contrast, the majority of the applications were task-oriented and tailored to specific subjects (e.g., biology), offering support for SRL within the context of particular learning tasks (e.g., writing). Different types of AI applications or tools provide varying levels of support for the SRL process. As illustrated in Fig. 3, all applications and tools predominantly influence the performance phase, particularly by facilitating monitoring and control. While evaluation systems and chatbots can play a role across all three SRL phases, their primary functions differ. Evaluation systems are more focused on the forethought and evaluation phases, assisting learners in planning and reflecting on their learning progress, whereas chatbots primarily support the performance phase by providing real-time interaction and guidance.

### Positive and negative aspects of AI applications for self-regulated learning

Each SRL phase was analyzed through both positive and negative perspectives (see Table 4 for details).

**Table 4:** Positive and negative empirical evidence of AI-empowered learning applications from the perspective of self-regulated learning

SRL phase		Positive aspects	Negative aspects
Forethought phase	Self-motivational belief	**[General]**: 1. Expressing a positive **attitude** toward the AWS system^17^ 2. More **motivated** to learn^15^ 3. Developing learning **self-efficacy**^18^ **[Specific]**: Enhancing **motivation** by offering up-to-data progress summary^20^	**[General]**: 1. Lack of familiarity with digital technologies results to **less enthusiasm** or **interest** in the AWE system^17^ 2. Lack (or **absence**) of **emotions**^16^ 3. Did not influence the improvement of the dynamics of **self-efficacy** for both effective and ineffective students^16^ **[Specific]**: 1. Click-based systems seem to be more helpful as a **low-threshold entry** point to coaching^33^ 2. Needed **more encouraging language**^20^
	Planning	**[Specific]**: Providing a clear roadmap for their studies makes it easier to **organize their time and tasks**^20^	**[General]**: Despite following recommendations, they couldn’t achieve their **set goals**^20^ **[Specific]**: Suggesting adding date and time functionalities to better **plan and manage** their study schedules^20^
Performance phase	Monitoring	**[Specific]**: 1. **Showing task priorities** (high, medium, or low) was praised for helping students understand what needed to be done to pass^20^ 2. Chatbot feedback types contribute greatly to students’ **metacognitive reasoning**^32^	**[General]**: The dashboard did not adjust to **reflect** their completed tasks **accurately**^20^
	Controlling	**[General]**: 1. A significant number of students demonstrated a high level of engagement by **adopting rewriting operations** in their revisions^17^ 2. Both difficulty and **mental effort** were significantly lower for the AI-enriched book^34^ 3. Demonstrated a high level of engagement suggests the use of **self-regulation strategies**^28^	**[General]**: The AI-based features did not answer basic (or all) posed questions, with some participants expressing a **need for more in-depth answers**^34^ **[Specific]**: The tool did not offer any useful guidance on **managing their study time**^20^
Self-reflection phase	Self-judgment	**[General]**: Participants appreciated the **guidance** on which aspects of their work required more focus to enhance their performance^20^	**[General]**: They expected a **calculated grade based on their activities**, which was not clearly communicated by the dashboard^20^ **[Specific]**: Grammarly does not **consistent**ly identify the same error depending on the text length and Grammarly’s **feedback** for structure is less consistently reliable than that for grammar^16^
	Self-reaction	**[General]**: Providing feedback on student performance, along with opportunities to repeat the “task-performance-feedback cycle” by allowing **resubmission**^19^	N/A

The following table summarizes the positive and negative aspects of the AI application in terms of its support for self-regulated learning (SRL) in the reviewed papers, based on Zimmerman’s three-phase SRL model. Each aspect within each process was categorized as either general or specific result.

In the forethought phase, AI applications positively impact the improvement of learners’ learning attitudes, motivation, and self-efficacy. However, improvements in learning self-efficacy were not consistent. For instance, students with initially low self-efficacy often struggled to leverage AI tools effectively. From the negative aspects, some learners’ lack of familiarity with digital technologies led to reduced interest and a diminished appreciation of the benefits of AI applications. Additionally, the absence of emotional engagement in AI tools was a common critique.

During the performance phase, AI tools were positively received for their ability to help learners organize their tasks, which significantly contributed to their metacognitive monitoring of learning progress. This assistance allowed students to spend less mental effort and to engage more actively in their learning behaviors, making necessary adjustments along the way. On the negative side, some studies reported that AI tools did not always accurately reflect the tasks at hand, causing confusion among learners. Additionally, there was a noted need for AI systems to provide more in-depth responses to students’ questions.

In the self-reflection phase, learners appreciated the feedback provided by AI applications, which guided them in improving their task performance. However, they also highlighted the importance of the accuracy and consistency of this feedback. Inaccurate or inconsistent feedback from AI tools was seen as a significant drawback.

## Discussion

Our systematic review of AI applications in supporting SRL has elucidated several key trends and gaps within the current body of literature.

Firstly, despite the burgeoning interest in leveraging AI to enhance educational outcomes, there is a notable paucity of research specifically exploring AI’s role in SRL, despite its status as a core skill for human learning. The limited number of studies is consistent with the challenges posed by the multifaceted nature of SRL, which demands sophisticated support mechanisms that are difficult to operationalize through AI technologies^7^. This gap underscores the nascent stage of integrating AI into this nuanced domain^11^. As each aspect of SRL emphasizes the multilayered characteristics of the learner based on their context^12^, the specific AI applications in SRL require further empirical exploration and validation^1^. Future research must broaden and deepen investigations into AI’s capacity to augment these intricate SRL processes and strategies, thereby advancing both theoretical and practical understanding in this field^8^.

Secondly, learning theories in the intelligence era should be expanded and restructured. Our review reveals a strong reliance on established educational theories in guiding the development and implementation of AI tools for SRL. Predominantly, studies are anchored in classical frameworks such as Zimmerman’s SRL model or cognitive load theory, demonstrating these models’ robustness and relevance in grounding new technological interventions^6,8^. However, this dependency on traditional theories highlights a significant gap, i.e., the absence of innovative theoretical constructs that can capture the dynamic and evolving interactions between AI technologies and SRL^13^. The evolving landscape of AI in education necessitates the development of new frameworks that more precisely address the unique affordances and challenges presented by AI in supporting SRL^2^.

**Fig. 4:**
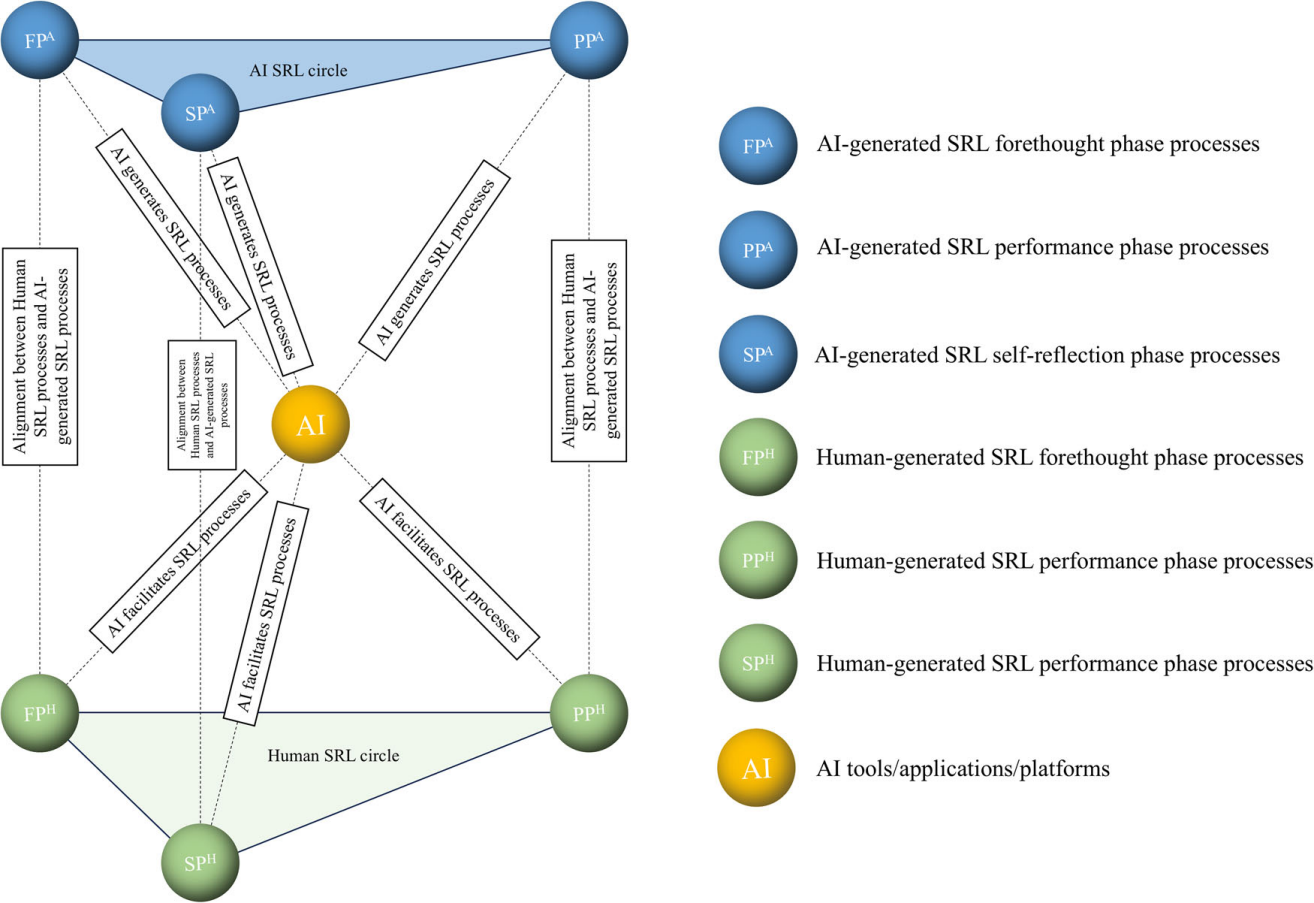
AI-Human interactive self-regulated learning model. In this model, with the AI support, there are human-centered SRL circle and AI-centered SRL circle. AI can propose expected SRL processes that are based on learners’ learning data, as well as provide tools or applications to facilitate SRL processes. Moreover, Human SRL processes and AI processes should be aligned based on human agency to effectively utilize AI to support their SRL.

In the application of artificial intelligence in education, it is essential to distinguish between human-centered self-regulation and AI-centered self-regulation. In human-centered self-regulation, AI serves as a facilitator by enabling learners to collect and access their learning data, providing intelligent platforms or tools that learners can use, monitor, and control, and supporting self-regulation through decision-making information generated by these platforms and tools^14^. In contrast, AI-centered self-regulation places AI in a central regulatory role, where it generates SRL cycles for human learners based on their data. However, the predominant form of SRL support should be regulation through human-AI interaction. AI must engage with the human-centered SRL cycle across multiple dimensions—such as behavior, emotion and affect, and cognition—to enhance coherence and ensure goal alignment between AI and human learners (see Fig. 4 for details). Novel theoretical developments are essential to provide nuanced insights and guide the optimal design and implementation of AI tools in this context^15^.

Thirdly, our analysis indicates that AI applications in SRL predominantly target specific phases of the SRL cycle rather than adopting a comprehensive, holistic approach. It is important to recognize that due to human-AI interaction, these applications or tools do not entirely disregard the overall coherence and continuity of SRL. Instead, they exert greater influence on certain phases while having a weaker impact on others. The AI-driven enhancement of these partial SRL processes can effectively address efficiency challenges and improve learning performance. Domain-specific or task-oriented applications tend to support learners’ strategies during the performance phase, e.g., Gridlock aids conceptual understanding and provides automated assessment^16^, Pigai offers collaborative feedback^17^, and Bashayer functions as a social AI assistant, delivering real-time feedback^18^. In contrast, tools that explicitly support general SRL processes, rather than being tied to specific subjects or tasks, tend to incorporate SRL holistically, e.g., Afzaal et al.’s explainable AI-based tool offers data-driven SRL support, while Rasa, a conversational AI, personalizes learning from the outset, guiding learners through goal setting, task execution, and self-awareness enhancement via feedback. However, this selective support highlights a crucial gap in fully leveraging AI to comprehensively support all SRL phases^19^. Since SRL emphasizes the consistency of goals, actions, and feedback, a lack of alignment may hinder the accurate evaluation of whether learning objectives are truly achieved. Future research, guided by our AI-Human Interactive SRL model, should further investigate the dynamic regulation of SRL within human-AI interactions.

Fourthly, although the majority of AI tools developed for student use offer significant benefits, the lack of applications specifically designed to assist educators in fostering SRL among their students remains a critical concern. Given that teachers’ self-regulation can ultimately influence students’ SRL^10^, this gap warrants further attention. This aligns with the broader implications discussed in our introduction, emphasizing the need for AI systems that not only empower students but also support educators in facilitating the complete SRL process^5^. The interaction between teachers and AI is inherently more complex, as teachers simultaneously function as learners, instructors, and managers. They must consider their content knowledge, pedagogical knowledge, and knowledge about technological integration within their teaching practices. Therefore, future research should focus on developing AI solutions that offer integrated support across the entire SRL cycle, ensuring coherence and alignment while enhancing collaboration with educators. For instance, AI could function as an intelligent agent to assist teachers in making more informed decisions regarding their students’ learning progress and instructional strategies. Furthermore, from a socio-ecological perspective, future research should explore AI-supported SRL not only at the individual level but also at the interpersonal and community levels. Investigating how AI facilitates SRL in collaborative learning environments, peer interactions, and broader educational ecosystems can provide a more comprehensive understanding of its impact. This multi-level approach will help uncover the dynamic interplay between AI, learners, educators, and learning communities, ultimately contributing to more holistic and effective AI-driven SRL interventions.

Finally, the review identifies both promising benefits and significant challenges associated with AI’s role in SRL. On the positive side, based on big data, AI can enhance SRL by offering personalized learning experiences and immediate feedback, which can increase student-sustained learning engagement and improve learning outcomes^16^. These advantages align with our introductory expectations regarding AI’s transformative potential in education^17^. However, the review also highlights potential drawbacks, such as the risk of learners becoming overly dependent on AI tools or experiencing feedback overload, which may hinder their agency to autonomously regulate their effective learning. For instance, even when AI provides recommendations and learners follow them accordingly, their learning goals may still not be achieved^20^. This could be attributed to the tendency of individuals to prioritize fast and seemingly optimal solutions over slower, more practical ones^21^. Such a preference may lead to superficial engagement with AI-driven guidance, potentially undermining the deeper cognitive and metacognitive processes essential for effective SRL. These findings suggest that while AI holds considerable promise, its implementation must be approached with caution to avoid exacerbating existing challenges in SRL. For instance, teachers should play a significant role in strategic instructional design that promotes critical engagement with AI technologies^22^. Educators and developers need to strike a balance between maximizing the benefits of AI and mitigating its potential risks, thereby fostering a supportive and sustainable integration of AI in SRL practices^23^.

## Methods

The selection of studies to be included in this study followed the preferred reporting items for systematic reviews and meta-analysis (PRISMA) statement^24^. PRISMA provides specific reporting guidelines on the essential components to be reported in each section of the review study^25^. Six explicit, transparent, and replicable steps were conducted accordingly: (1) specification of eligibility criteria; (2) specification of information sources; (3) specification of search strategy; (4) study selection; (5) data extraction; (6) content analysis. To facilitate the review, a qualitative analysis tool, MAXQDA^26^, and Excel were used.

### Step 1: Eligibility criteria

This systematic review will include studies that focus on higher education settings. To be eligible for inclusion in this review, studies must involve the use of artificial intelligence (AI) technologies designed to support or enhance learning processes. This encompasses a broad range of AI applications, including intelligent tutoring systems, adaptive learning platforms, and AI-driven feedback mechanisms, all of which are aimed at fostering SRL. In order to ensure the inclusion of robust and evidence-based findings, only empirical studies employing either quantitative, qualitative, or mixed-methods approaches will be considered. This review aims to collate comprehensive and high-quality insights into how AI technologies are being employed to promote SRL within higher education contexts.

### Step 2: Information sources

The databases services of Web of Science, Scopus, EBSCOhost (including Academic Search Complete, Business Source Complete, British Education Index, Communication & Mass Media Complete, Criminal Justice Abstracts, CINAHL Plus, Educational Resource Information Center (ERIC), MEDLINE), and ProQuest (including Australian Education Index (AEI), PsycARTICLES, PsycINFO, Sociological abstracts, Education Database) were searched for relevant studies from their respective inception dates to June 7, 2024. Google Scholar was applied to facilitate the snowballing search^27^.

### Step 3: Search strategy

Based on the database services, we limited the search to peer-reviewed English-language journal articles. The search fields targeted were the article title, abstract or keywords. To search for relevant articles, three sets of key terms were applied for the initial search:

1. “artificial intelligence” OR “machine learning” OR “intelligent tutoring systems” OR “adaptive learning technologies” OR “learning analytics” OR “educational data mining”
  AND
2. “self-regulated learning” OR SRL OR metacognition OR “self-monitoring” OR “self-assessment” OR “goal setting” OR “self-reflection” OR “learning strategies” OR “autonomous learning”
  AND
3. “higher education” OR “university education” OR “college education” OR “postsecondary education” OR “tertiary education”

### Step 4: Study selection

The study selection process was guided by the four-stage PRISMA flow diagram^24^, which consisted of identification, screening, eligibility, and inclusion. In the identification stage, all search results were exported to Excel and duplicates were removed. Next, the titles and abstracts of all articles were screened against the eligibility criteria. The studies were removed based on the established criteria:

- Learning context was not in higher education.
- The study was not an empirical study with data collection and analysis.
- The study did not investigate the impact of AI applications for SRL.
- The study did not involve any description of how AI application was used.
- The study was not published in a peer-reviewed English-language academic journal.

The remaining full-text articles were then examined further to determine their inclusion statuses. The study screening process was performed independently by the two authors. We discussed and resolved all discrepancies. In addition, forward-searching and backward-searching were conducted on these included studies. Forward searches attempt to find other studies that had cited the included studies, and backward searches attempt to find other studies that were cited in the included studies^27^. We conducted forward searches by using the “cited by” function below the article record in Google Scholar and did backward searches by checking the references list in each included article. Forward and backward searches were carried out with those newly found, relevant articles until no more relevant article could be obtained.

### Step 5: Data extraction

Each study’s basic information (i.e., the aim of the study, research design, results summary), theoretical foundation (i.e., theoretical or pedagogical framework), and AI application (i.e., what and how AI application was used, and underlying backend technology) were extracted (See Table 5 for coding schema).

**Table 5:** Coding schema

Dimension	Code	Explanation
Demographic information	The aim of the study	Briefly summarize the objective of the reviewed study.
	Research design	Summarize the research method, research participants and sample size, data collection and analytical approaches, and location of the study.
	Brief results	Briefly summarize the empirical evidence of the study.
Theoretical foundation	Theoretical framework	The theories that can explain how learning is processed. For instance, cognitive load theory explains how cognitive load can impact learning effectiveness.
	Pedagogical framework	The theories that can inform how a learning process can be designed. For instance, team development provided step-by-step guidelines on instructional design. Learning strategy such as learning by doing is also counted as the pedagogical framework.
AI application	What AI application used	Describing what AI application have been used in the study.
	How AI applications used	Describing how AI application has been applied in detail in the study, including the process and activities etc.
	Backend technology	Describing what AI technology has been used underlying the AI application.

The following table delineates the dimensions and codes that were utilized in the process of extracting the reviewed papers. Each code was elucidated by definition or through the presentation of pertinent examples.

### Step 6: Content analysis

In analyzing the integration of artificial intelligence (AI) tools in higher education through the lens of Zimmerman’s SRL theory^11^, it is essential to first summarize the foundational aspects of this model. Zimmerman’s SRL theory posits that learning is an active, constructive process where learners set goals, monitor, and regulate their cognition, behavior, and motivation. This model delineates SRL into three interrelated phases: forethought, performance, and self-reflection. During the forethought phase, learners engage in task analysis and motivational beliefs, setting the stage for strategic planning and goal setting. The performance phase involves implementing learning strategies and monitoring progress. Finally, the self-reflection phase encompasses self-assessment and adjustments based on feedback and outcomes.

Connecting these phases to the functionalities of AI tools, we observe diverse applications that support SRL at each stage. AI-driven platforms such as adaptive learning systems aid in the forethought phase by providing personalized learning paths and goal-setting frameworks tailored to individual learner needs. During the performance phase, intelligent tutoring systems and AI-based analytics offer real-time feedback and adaptive support, enabling learners to apply effective strategies and track their progress. For the self-reflection phase, AI tools like learning analytics dashboards and NLP-based feedback mechanisms facilitate in-depth self-evaluation and promote reflective practices by analyzing performance data and suggesting areas for improvement.

The empirical evidence of AI’s impact on SRL within higher education can be categorized across these three phases, revealing both positive and negative outcomes. Positively, AI tools have been shown to enhance forethought by improving learners’ goal-setting and planning capabilities through customized pathways and predictive analytics. In the performance phase, AI applications often lead to increased engagement and strategy use by providing timely and tailored feedback. As for the self-reflection phase, AI-driven insights and reflective prompts have been linked to improved self-assessment accuracy and deeper learning reflections. However, there are also negative aspects to consider. In the forethought phase, over-reliance on AI for planning can lead to reduced learner autonomy. During performance, the potential for feedback overload or dependency on AI for guidance can undermine self-efficacy. In the self-reflection phase, there is a risk that AI-generated feedback may not always align with learners’ internal reflections, potentially leading to disengagement or confusion.

This analysis underscores the nuanced role of AI in supporting SRL within higher education and highlights the importance of balancing technology with learner autonomy and engagement across all phases of SRL.

## Supplementary information

Reviewed paper summary table

## Data Availability

Further data are available upon request. The source data, i.e., the reviewed papers, are provided in the references with marks. The summarized data is presented in Table 1.
